# Reframing knowledge translation for health policy in Kenya: actors, practices and the constitutive role of context

**DOI:** 10.1186/s12961-026-01482-5

**Published:** 2026-04-19

**Authors:** Fatuma Hassan Guleid, Edwine Barasa, Gilbert Abotisem Abiiro, Jacinta Nzinga

**Affiliations:** 1 Health Economics Research Unit, KEMRI-Wellcome Trust Research Programme, 197 Lenana Place, Lenana Road, Nairobi, Kenya; 2 Center for Tropical Medicine and Global Health, Nuffield Department of Medicine, University of Oxford, Oxford, UK; 3 Department of Health Services, Policy, Planning, Management and Economics, School of Public Health, University for Development Studies, Tamale, Ghana; 4 Liverpool School of Tropical Medicine, Liverpool, UK

**Keywords:** Knowledge translation, Health policy

## Abstract

**Background:**

Knowledge translation (KT) is regarded as important for supporting evidence-informed health policy-making. While KT models have moved beyond linear understandings of the evidence–policy relationship, they continue to underplay the politics that shape how evidence is produced, framed, interpreted, negotiated and used during policy-making. As a result, KT strategies are often designed for policy processes that bear little resemblance to real-life policy-making.

**Methods:**

This study argues for a reframing of KT for policy as an embedded and politically situated process. To inform this reframing, we examined how KT happens during health policy-making in Kenya. We examined who engages in KT, how they do it, in which spaces and the outcomes of these practices. Data were collected through in-depth interviews with a range of policy actors (*n* = 35), nonparticipant observations (52 h) and document reviews (*n* = 34). Data analysis was informed by the study’s conceptual framework.

**Results:**

The findings show that KT was enacted by a range of actors, including policy-makers themselves. These actors practised both so-called structured and fluid forms of KT and mobilized evidence to inform, advocate, justify or contest policy positions. In addition, KT happened in both formal and informal spaces. Strategic framing of evidence and other relational activities were central to mobilizing evidence. The outcomes of these practices were often relational and incremental. Importantly, context constituted KT by shaping what counts as evidence, whose voices were influential, and where action was possible.

**Conclusions:**

This study offers a practise-based understanding of KT by reframing it as a contextually-constituted, situated practice that requires adaptive system-oriented approaches.

**Supplementary Information:**

The online version contains supplementary material available at 10.1186/s12961-026-01482-5.

## Background

Over the past two decades, knowledge translation (KT) has become dominant for addressing the gap between research, policy and practice. Often touted as a solution to this gap, KT has been widely adopted to support health systems strengthening and has become central to evidence-informed policy agendas globally [1–4].

However, KT frameworks, particularly those designed to guide practice, have often approached the relationship between evidence and policy in largely technical terms. Much of the practice remains focused on efforts to improve the communication and dissemination of evidence without interrogating the highly politicized context of policy-making characterized by negotiation, contestation and the pursuit of multiple and often-conflicting goals [5, 6]. Even more recent KT approaches that emphasize nonlinear processes and advocate for participatory approaches, such as integrated KT (iKT), tend to focus on engagement and coproducing research to improve relevance, without addressing the underlying assumptions about how evidence travels in policy spaces [7–9]. This dominant framing of KT has generated a host of conceptual models, toolkits and strategies to guide KT practice (many of which are now embedded in institutional practices [10]). While such efforts have increased attention to the importance of engagement between the research and policy communities, they also overlook the institutional politics, power dynamics and interests that shape how evidence is (or is not) used in policy contexts. In doing so, KT literature has inadvertently reinforced a technocratic view of policy-making that underestimates the role of power, values, institutional structures and epistemic hierarchies [11].

Policy-making is recognized as taking place within contested spaces in which multiple actors, values, institutional norms and political interests interact and where evidence claims and legitimacy are contested [12, 13]. This has been well documented in fields such as policy studies, science and technology studies (STS) and research on evidence use in policy-making. Several scholars have drawn from these fields to expand our understanding of KT in policy settings [5, 14–18]. Their work provide insights into the inherently political and situated nature of evidence and policy-making, suggesting that KT is a deeply social and political process of negotiation, meaning-making and interests alignment. However, while this scholarship has made significant theoretical contribution to the field of KT, uptake in KT practice remain limited. If KT is to meaningfully support evidence-informed policy-making, then it is important to better understand how KT actually happens in practice including who engages in it, which and how evidence is mobilized, how it is used and under what conditions [19]. This is especially important in low–middle income countries (LMICs), where policy systems are often characterized by the influence of global agendas [20–23]. This study responds to this gap by examining the KT for health policy-making landscape in Kenya to contribute to a more grounded and context-sensitive understanding of KT processes.

By situating KT within its real-world political, institutional and social contexts, this study contributes to KT scholarship by suggesting an empirically grounded reframing of KT as a diffuse, situated, political and social practice that is constituted through context. This provides a practice-based understanding for the design of more policy-making attuned KT strategies.

## Methods

### Conceptual framework

We developed a conceptual framework informed by the field of policy studies, STS and evidence-use literature. We drew pragmatically from these fields to expand the scope of KT. For example, we incorporated ideas such as actor coalitions, power, political interests, institutional logics and policy-change dynamics from the field of policy studies [24–26]. We used concepts from STS that highlight evidence as socially constructed, including concepts from coproduction and boundary work to understand the socially constructed nature of evidence and how credibility, authority and legitimacy are produced and negotiated [27–31]. Lastly, we drew insights on how evidence is filtered and used or ignored within policy settings borrowing from evidence-use models [32]. The resulting framework aimed at moving beyond the technical and instrumental conceptualization of KT by highlighting the political and situated nature of KT (Table 1).

**Table 1:** Theoretical constructs

Construct	Conceptualization
Actor	Heterogeneous, relational and agentic. Policy theories (for example, advocacy coalition framework, policy network theory) show that actors operate as members of coalitions and networks; STS extends this by showing that actor authority, credibility and legitimacy is actively produced through boundary work and through enrolment in socio-technical networks (actor network theory). Analytically, this directs attention to coalition alignment, boundary maintenance and the material supports that enable or constrain actor voices. Actors practice KT by producing, mobilizing and using evidence in ways that align with their interests, roles and capacities
Evidence	Defined broadly to include both explicit (for example, research, reports etc.) and tacit knowledge (for example, experiential evidence). Policy theories emphasize that ideas shape what is considered evidence; STS suggests that evidence is materially and socially enacted and coproduced through instruments (for example, policy briefs), infrastructures and practices. Thus, evidence is both epistemic content and a product of material-social work (that is, how it is generated, represented and mobilized matters for its policy use)
Context	The institutional, political, social and epistemic conditions under which KT occurs. It is treated as constitutive rather than merely moderating (coproduction). STS proposes that context is produced in conjunction with knowledge: material infrastructures (data systems, models, guidelines), meeting routines and boundary objects create and reproduce the contextual conditions that render evidence intelligible and actionable. Context, for example, institutional arrangements, structures the resources, norms and expectations that shape actors’ capacities to engage with evidence. In turn, actors reconfigure context through their everyday practices for example, mobilizing networks, framing problems and so on (actor-centred institutionalism)
Processes	Activities/strategies/interventions through which evidence is mobilized: framing and narrative construction (multiple streams framework, narrative policy framework), coalition-building and long-term advocacy (advocacy coalition framework), institutional navigation (actor-centred institutionalism, 3-Is (interests, ideas and institutions) and the STS forms of translation (enrolment, boundary work and coproduction of problems and solutions). These processes are not neutral activities; they are political acts that reconfigure who counts, what counts and how issues are prioritized. KT processes mediate between evidence, actors and context
Outcomes	Plural and temporal. Building on Caplan and Weiss, the framework differentiates instrumental, conceptual and symbolic uses and also recognizes material and institutional outcomes (new procedures, platforms, routines). Crucially, outcomes must be evaluated against time: conceptual influence accumulates; symbolic use can legitimate pre-existing choices; instrumental uptake is frequently contingent on windows of opportunity (Punctuated Equilibrium Theory/Multiple Streams Framework) and coalition power (Advocacy Coalition Framework). The outcomes of KT, whether instrumental, conceptual or symbolic, feed back into contexts, reshape actor roles and recalibrate what counts as evidence

This framework informed the study’s design and analysis. It was used to develop interview guides and observational protocols, ensuring attention to these constructs during data collection. For analysis, the constructs served as guiding concepts during coding, theme development and interpretation (Table 2).

**Table 2:** Operationalizing the framework (non-exhaustive)

Construct	Subconstructs	Example analytical questions
Evidence	Framing and definition of evidence	How is evidence defined, and framed and by whom?
	Source and credibility	What sources are considered legitimate or authoritative? Why?
	Role	What role does evidence play (to justify, persuade, delay)?
Actors	Actor roles and identities (shifting or fixed)	Who are the key actors? How do they understand their roles? How do institutions shape these actor roles?
	Relationships (networks/coalitions)	What types of relationships exist? How are they formed and sustained? How do they influence KT?
	Power (expertise, positional authority, relational capital); interests	What forms of power do actors draw on in the KT-policy process and how do they exercise them? Where is power exercised? What is its effect?
Context	Institutional structures, mandates, rules, cultures, capacity	What institutional arrangements, cultures and norms shape KT? How?
	Normative and ideational environment	What structures, norms and ideas shape what counts as evidence?
	Policy context	What political dynamics shape KT? How? How does the policy issue affect KT?
KT processes	Problematization (framing issues and agenda setting)	How are problems framed, and what practices are used to get them onto the agenda?
	Boundary work (boundary spanning, boundary objects)	What boundaries are being drawn, and who manages them? How are boundaries crossed?
	Selection, framing, interpretation, legitimation, packaging and circulation of evidence	How is knowledge moved, framed and negotiated across actors and settings?
	Negotiation and brokerage	How do actors align and gain buy-in?
KT outcomes	Conceptual shifts (changes in how problems/solutions are understood)	What types of outcomes can be observed beyond policy change?
	Relational effects	How does KT reshape relationships, trust, credibility and actor configurations?
	Instrumental effects (policy or programmatic change)	How do actors use KT to signal legitimacy or build credibility?
	Symbolic use (to signal alignment or legitimacy)	How, why and when is evidence used symbolically?

### Study context and design

We used a cross-sectional qualitative research design to examine how KT is practised within Kenyan health policy-making. This study was conducted at the national and subnational levels in Kenya. Following adoption of the 2010 Constitution, Kenya underwent a major decentralization reform that created 47 semi-autonomous county governments with responsibilities for health service delivery [33]. The national government, through the Ministry of Health (MoH), retained control over policy formulation, training and regulation. Policy-making occurs through a multilevel governance structure, with both national and county governments playing roles in health policy [33]. This restructuring has introduced county governments as new spaces and actors for health policy-making [34]. Kenya presents an interesting setting for studying KT for two main reasons. First, Kenya’s health sector has been marked by recent and active reforms which were politically motivated and embedded in electoral campaigns. This includes roll-out of universal health coverage (UHC), health insurance reforms and efforts to strengthen primary health care (PHC) [35]. These reforms are driven by a mix of political priorities, donor influence and global health agendas [36]. Importantly, they also serve as key sites of evidence production, policy experimentation and actor negotiation. For example, UHC pilots created space for policy experimentation where the learnings of these pilots were used to shape national scale-up plans while also revealing the tensions between national and county governments over roles and authority [36–38]. Second is its parallel efforts to institutionalize evidence use for health policy-making. Kenya has a vibrant ecosystem of research institutions and technical and development partners that routinely engage with policy-makers [39]. Within the MoH, there are KT structures, such as a unit mandated with supporting KT, intended to institutionalize evidence use. In addition, several county governments have also established research units within the department of health. However, the impact of these are yet to be evaluated.

### Case selection

We adopted a breadth-oriented approach to selecting KT policy-making cases to study. The initial scope of the study focused on broad health system reforms over the past 3–5 years that were relevant to both national and subnational contexts (for example, UHC, PHC). As the study progressed, the scope of focus evolved to include policies that participants themselves referenced as particularly salient to the study questions. These included autonomy reforms at the county level, reproductive health and disease-specific policies such as malaria control policies. While this broad approach mean that we could not study each case in depth, it enabled us to explore the complexity and context-dependent features of KT in policy processes.

### Study population and participant selection

The study population included participants working at both national and county levels. Three counties were selected purposively to reflect institutional diversity and varying relationships with research and evidence use. County A has a longstanding institutional partnership with a major health research institution and is recognized as a national learning site for health systems research. It has formal structures that explicitly incorporate research into decision-making, making it a useful site for understanding institutionalized KT practices. County B demonstrates an openness to collaboration with researchers and development partners and has implemented major reforms, including universal health coverage (UHC) initiatives, with notable levels of public participation. This county provided insights into how technical expertise and external partnerships shape KT processes. County C is more geographically remote and recently legislated and implemented a key autonomy reform. While it receives substantial support from development partners, it lacks visible or institutionalized mechanisms for engaging with evidence. Its inclusion allowed the study to explore how KT unfolds in less structured environments and to contrast with more research-active counties.

The study participants included the policy-makers from the MoH and county department of health (CDoH), researchers/academics, intermediaries, development partners and other key actors (Table 3). The study focused on actors engaged in the practices through which evidence is produced, circulated and used. While politicians and political leaders shape the political direction of policy, they are less directly involved in these processes. To capture the parliamentary interface with evidence, we included the parliamentary research office that supports the Parliamentary Health committee.

**Table 3:** List of participants interviewed

Study participants	Number
Category	
Policy-makers (MoH and CDoH)	17
Policy advisor	1
Semi-autonomous government agencies	1
Researchers/academics	8
Intermediaries/knowledge brokers	3
Development/implementation partners	4
Advocacy	1
Total	35

We employed a purposive and snowballing approach to identifying and selecting participants. Purposive sampling allowed us to include participants who had direct experience with KT and policy-making. To complement this approach, we used snowballing to identify additional participants that we may have overlooked in our initial sampling.

### Data collection

Data were collected between January 2024 and February 2025 through three qualitative methods: in-depth semi-structured interviews, nonparticipant observations and document reviews. These methods were selected to enable a rich and triangulated understanding of how knowledge translation (KT) happens in practice across different spaces and actors.

A total of 34 interviews were conducted. Interview guides were developed using the study’s conceptual framework, ensuring coverage of key domains such as how evidence is understood and mobilized, actor roles and relationships, contextual constraints and KT practices and outcomes (Additional file 1: Supplementary Table 1). The guides were piloted and revised where necessary. During the interviews, we remained flexible to allow participants to define concepts such as evidence and KT in their own terms. Interviews lasted 30–60 min and were audio-recorded, transcribed verbatim and conducted at times face-to-face or virtually.

In addition to interviews, 52 h of nonparticipant observations were carried out across a range of KT policy-relevant events including technical working group meetings, stakeholder consultations and evidence-sharing forums. Detailed field notes captured not only content but also interactional and relational dynamics, enabling insights into informal forms of KT, framing and actor negotiation (Additional file 1: Supplementary Table 2). Document reviews (*n* = 34) included policy guidelines, strategy documents, meeting minutes and internal memos relevant to the KT processes under study. These materials were selected to complement interview and observational data and to help trace the institutional narratives and evidence use practices over time. Documents were selected on the basis of their relevance to the observed processes and actors interviewed. All data collection was iterative, with emerging insights informing subsequent interviews and observations (Additional file 1: Supplementary Table 3).

### Data analysis

Data analysis was done following a framework analysis using NVivo software (FHG and JN). We combined both deductive and inductive coding, guided by the study’s conceptual framework. First, we familiarized ourselves with all the data, during which we wrote initial memos to capture early impressions about the data. Then, we used inductive coding to generate initial codes in an open and exploratory manner. This was followed by deductive coding drawing from the conceptual framework to refine and expand our coding structure. Combining inductive and deductive coding at this point allowed us to pay attention to both analytical dimensions (for example, institutional structures) in the framework and new insights not covered in the framework (for example, embedded practice). Next, we collated the codes into broader themes to capture patterns across the data. For example, codes related to informal consultations, technical working groups and dissemination forums were grouped under the theme called KT spaces. Thereafter, we reviewed the themes against the full data set and reinterpreted them using theoretical lenses including coproduction, boundary work and so on. This iterative approach led to refining and collapsing overlapping themes to ensure that each theme was analytically coherent and well supported across the entire dataset. We then defined and named themes on the basis of their contribution to understanding how KT unfolds in practice.

## Results

### Who is doing KT and why? Actors and their motivations

Across Kenya’s health policy landscape, KT is enacted by a heterogeneous set of actors situated within institutional, professional and epistemic communities. These actors constitute overlapping networks that converge around specific policy problems, funding opportunities, or reform initiatives (Table 4).

**Table 4:** Actors in the Kenyan KT health policy space (non-exhaustive)

Actor category	Examples	Role in policy	Involvement in KT
Politicians and elected officials	Members of parliament, senators, governors	Allocate health budgets, pass health laws	Make decisions, advocate for health reforms; leverage evidence for political agendas
Bureaucratic Actors	Ministry of Health (MoH), county department of health (CDoH)	Formulate policies, implement programs	Use evidence for decision-making; commission evidence; coordinate policy processes; stakeholder engagement; generate evidence and data; advocate for health issues
Semi-autonomous government agencies (SAGAs)	Kenya Medical Research Institute (KEMRI), Kenya Health Professions Oversight (KHPOA)	Bridge policy and implementation by providing specialized technical expertise, managing health resources, service delivery, quality assurance, regulation and so on	It depends on the specific SAGA. Involve generating/disseminate evidence; provide advice; implement national policies
Regulatory bodies	Pharmacy and Poisons Board (PPB)	State and enforce standards, ensure compliance and safeguard quality	Generate/disseminate evidence; provide advice; implement national policies
Professional councils	Kenya Medical Practitioners and Dentists Council (KMPDC), Nursing Council of Kenya, Clinical Officers Council	Set training/licensing standards; ethical oversight	Generate/disseminate evidence; advise government; develop clinical guidelines; advocate for workforce policies
Academia and research	Universities, research institutions	Conduct health research, provide technical advice	Generate/disseminate evidence; advise policy-makers; build capacity for evidence use, advocate for evidence use
Intermediaries/knowledge brokers	The African Institute for Development Policy	Build EIPM capacity policy-makers; facilitate multi-stakeholder dialogues	Synthesize evidence; build KT capacity
Donors and development/implementation partners	WHO, PATH, Clinton Health Access Initiative, World Bank	Fund programs, fund research, provide technical assistance, support implementation	Commission research; fund research; push for data-driven policies; support health information systems; convene stakeholders; support implementation
Unions and associations	Kenya Medical Association (KMA), Kenya Union of Clinical Officers (KUCO)	Advocate for health workers’ rights, strike for better policies	Disseminate evidence; use workforce data to advocate for staffing norms, salaries and safety standards
Civil society (CSOs)	LVCT Health, Kenya AIDS NGOs Consortium	Advocate for communities and marginalized groups; monitor service delivery	Generate and disseminate evidence; advocate for community needs
Media	Health journalists	Report on health issues, scrutinize policies, publicize research	Simplify technical health data for public awareness; amplify expert voices

Researchers often enacted KT as a discrete, project-bounded activity that was planned for. In this framing, KT was structured, episodic, temporally sequenced and often tied to tangible outputs like policy briefs, dissemination events and so on (so-called structured KT). However, several researchers, particularly those who had sustained engagement in policy processes or who occupied hybrid roles (both policy and research roles), practised KT in more embedded and relational ways.

“I was particularly involved in the thinking process to fine-tune it and so forth and think through the elements that goes on into it [community health strategy].” Researcher 008

The forms of KT were shaped by the incentives and motivations of these actors. Researchers operating under the logic of accountability within the research-funding system, employed structured KT to share their research, demonstrate the relevance of their findings and fulfil obligations to translate research into policy:

“Part of that [coproducing evidence with policy-makers] was also driven by [Funder], I guess, you know, like, they had forced you to say how you’re working with government. So, sometimes it comes from also the funder, because of this global push to ensure there’s integration with policy makers.” Researcher 008

Conversely, other actors, such as policy-makers and development and implementation partners, enact KT as part of ongoing, embedded problem-solving. Their KT practices were integrated into their everyday work (what we will henceforth refer to as fluid KT). This form of KT was often pragmatic, tacit, immediate and not explicitly labelled as “KT” even though they involved the synthesis, exchange and application of evidence. Evidence was sought as needed, often from trusted sources rather than formal studies, and used to support negotiation, persuasion or justification:

“For the facility improvement fund (FIF) [bill], you present your budget [to the county assembly]. Funds allocated versus how funds are consumed. You bring out the approved budget and annual work plans and you are able to pick up obvious gaps in terms of funding. You also show patterns of availability of essential drugs and how that has affected service delivery. You also present case studies including particular cases…. All that is evidence that provides justification and push the case [for FIF].” County Policy-maker 001

Furthermore, we found that development/implementation partners, who often spanned multiple institutional roles, engaged in both structured and fluid KT. For example, they practised fluid KT by embedding technical advisors in the MoH, coproducing data with policy-makers and supporting real-time decision-making. Here, KT was described as part of maintaining policy momentum and responsiveness. At the same time, they also engaged in structured KT by producing and disseminating knowledge products such as policy briefs, technical reports, hosting high-level briefings and so on. These practices were often aimed at advocacy, agenda setting or steering policy direction:

“When we are advocating for a county to, for example, come up with a budget line that has not been there, we will be presenting evidence of what has worked in other contexts [saying] ‘this is the impact of this budget line’.” Advocacy partner 007

These findings suggests that KT practice is diffuse and distributed amongst actors and organized around institutional logics and roles. Finally, the varied purposes reveal how KT often extends beyond informing policies and instead supports multiple actor interests and functions across policy-making. A more detailed account of the role and influence of actors in shaping KT policy process is published elsewhere [19].

### Where is KT happening? Spaces and structures

KT actors’ approach and motivation for doing KT in turn shaped where KT happened. Formal spaces, often utilized when practising structured KT, included structured settings such as policy advisory groups, dissemination meetings and stakeholder meetings where evidence was shared or debated. These were typically initiated through official channels and involved scheduled, time-bound engagements. While such settings offered important opportunities for engagement, we observed that access to them was highly regulated and limited to actors with institutional legitimacy and authority, funding relationships and relationships with the conveners of these spaces.

However, KT also happened in informal spaces, such as corridors, personal phone calls or WhatsApp discussions, especially where strong interpersonal relationships between actors already existed. These informal spaces were critical for problem-solving, clarifying evidence needs or aligning perspectives outside the constraints of formal procedures:

“We’ll often meet the decision-makers…on the side, you know, for tea, coffee, phone calls to also really understand what different people’s agendas are so that you know, when we’re all together there in the big boardroom, you have a better understanding of who wants what”. Development partner 008

We also observed that, in practice, actors often moved between formal and informal spaces, and the same space could serve different purposes depending on the actors’ goals for KT.

These distributed spaces for KT highlight the limitations of assumptions of demarcated or structured points/spaces for evidence exchange. It also indicates that KT spaces are produced through institutional and relational structures of the policy ecosystem which mediates access to these spaces. This highlights the importance of examining not only the practices of KT but also the spaces where KT is practised and how they influence access and openness to deliberation.

### How is KT done?

Across the cases, KT practices included generating and synthesizing evidence, framing it for relevance, engaging in exchange, mobilizing champions and institutionalizing routines for evidence use. Fluid and adaptive practices were embedded in everyday policy-making work (fluid KT). These practices involved iterative exchanges, sense-making and negotiation (for example, evidence framing, coalition building, informal advice and so on). Evidence was mobilized opportunistically or responsively during meetings or through personal relationships. Importantly, both structured and fluid KT were enacted in tandem (although not always equal in measure or visibility).

Actors employed some specific practices that shaped how evidence was interpreted and made actionable. This included framing of evidence and coalition-building. For example, one participant working on developing a quality-of-care rating system for health facilities in Kenya described how initial engagement with policy-makers led to disagreements on how to rate facilities. However, upon conducting a pilot study and reframing the evidence, they were able to facilitate agreement and move the decision-making process forward:

“..when we presented these results [on quality in facilities], we basically told them that if you take the all-or-none [rating scale], where you give somebody zero even if they are missing a thermometer, then you’ll have to close nearly 90% of all health facilities in Kenya. That helped them [regulators] make a decision very fast. They actually said the political backlash from that would be too high.” Researcher/national policy-maker 005.

This kind of adaptive response involves determining what kind of evidence is needed in the moment, producing it (if necessary) and shaping it to fit the decision space. It reflects KT practice that is attentive to political and practical constraints. Such KT practices were more commonly practised by actors engaged in fluid KT, where KT was not a distinct activity but part of the everyday work of navigating uncertainties, relationships and policy-making. They often required proximity to the policy process, responsiveness and strategic flexibility (conditions that structured KT alone may not provide).

What emerges from these findings is a view of KT as a strategic set of practices oriented toward making evidence usable and persuasive, involving both technical skills and relational judgement and often requiring adaptability and proximity to policy.

### What evidence is used and how?

#### Evidence types and their role in policy-making

Participants consistently engaged with different types of evidence during the policy process (Table 5).

**Table 5:** Types of evidence used by Kenyan health policy-makers

Evidence type	Example source(s)	Role (non-exhaustive)	Perceptions
Routine national and county health data	KHDS, MoH reports	Monitoring trends, planning and prioritization	Timely, locally owned, widely accessible
Research evidence	Peer-reviewed journals, dissemination by researchers	informing interventions, legitimizing choices	Perceived as credible, rigorous but less timely and not always relevant and accessible
Global guidelines and evidence	WHO, World Bank, other international organizations	Agenda-setting, justifying interventions, framing priorities	Seen as authoritative
Expert opinion	Technical advisors, experienced peers	Interpreting evidence, guiding choices	Rapid, context-sensitive, trusted
Evaluation reports	Program evaluations, program reports	Learning from past efforts, justifying scale-up	Practical insights, problem-solving
Operational data	Facility-level records, budgets, HRH data, supply chain systems	Managing service delivery, adapting programs, informing policy tweaks	Real-time, context-specific, supports practical decision-making

These types of evidence played different roles and were often used in combination, depending on the policy question and the policy stage. For example, scientific evidence, including findings from randomized control trials (RCTs), were most valued when policy-makers needed to address highly technical issues (for example, vaccine roll-out) or to measure effectiveness of interventions during policy formulation. By contrast, for issues such as managing human resources for health (HRH), policy-makers relied more on experiential knowledge, particularly from individuals managing HRH systems. This type of evidence was valued for its practical relevance and specificity to health worker needs:

“When it comes to HRH management also there are experiences. You’ll tap on the experiences more and reports more than a quantitative database, you know? The senior level managers. The people who are managing people because that is HR.” National policy-maker 001

Another widely reported type of evidence was what policy-makers referred to as “benchmarking”, where they drew ideas and lessons from other settings (both domestic and international) to inform their policy-making. Benchmarking enabled access to richer, more practical forms of evidence that were often perceived as more immediately applicable. For examples, counties that wished to develop facility autonomy reforms travelled to counties that had implemented these reforms to learn from their experiences.

Consistent in these findings (at both levels of government) is the primacy of practical and problem-solving evidence, suggesting that the value of evidence is in its utility:

“Policy-makers just need something that is pointing towards providing a solution to the question they have.” National policy-maker 007

Such pragmatism in selecting evidence types helps explain why operational data, for example, HR data or facility level records, often generated by the MoH and counties themselves, were the most frequently mentioned types of evidence used. These types of evidence were more timely, accessible and relevant to policy-makers.

### How is evidence used?

We observed that evidence was routinely used by policy-makers during policy deliberations, agenda-setting, problem-solving and negotiation processes. How this evidence was used varied depending on the actors involved, their motivations and the policy need.

For example, to inform prioritization and planning, evidence was used instrumentally, helping policy-makers identify where to focus policy attention and resources:

“We did a health labour market analysis which we are able to show where we are at, where we want to go, what it will entail to be able to do that. And looking at the magnitude of what we want to do, it cannot be achieved without having a policy in place.” National policy-maker 001

Such instrumental use was enabled by institutional alignment for example, the data fit within existing planning and budgeting frameworks. The mechanisms here were practical and procedural, that is, evidence was usable instrumentally when it addressed defined administrative needs and could be translated into concrete actions.

Symbolic use, where evidence served to defend or rationalize decisions that had already been made or to provide a credible basis for particular positions (legitimation and justification), was also observed/reported. In such instances, research was especially valued because of its perceived objectivity and rigour, lending legitimacy to decisions in the eyes of other actors. This points to the symbolic function of research evidence where it was not always about guiding choices but about demonstrating that decisions were informed, reasonable and defensible:

“Now that’s where it becomes scientific. Because it has to be evidence-based. You can’t be in something, and you do not have anything to support it. So, unless you provide evidence towards that…. that’s how you may be able to tackle that [interests].” National policy-maker 003

Evidence was also used symbolically for persuasion and advocacy, particularly when policy-makers needed to build coalitions or convince others to support a given policy direction. This highlights how evidence plays a role in navigating political landscapes and aligning interests:

“And also, for the level of the executive, using facts to advocate to them so that our needs can be on their agenda” County policy-maker 002

Symbolic use thus functioned as a type of “boundary” practice: it linked bureaucratic decisions to the normative authority of science, even when the connection was more rhetorical than substantive. The mechanism here was one of legitimation by invoking evidence as a performative signal of rationality and accountability in a system where such signals carried political value.

These findings indicate that the types of evidence used and how they are used is shaped by actors’ goals, institutional logics, politics and the nature of the policy issue itself. Evidence was used not simply to inform, but to position, justify and persuade. The use and non-use of evidence were themselves key practices through which actors navigated power, responsibility and legitimacy in the policy process.

### Context as constitutive of KT

#### Institutional context

The institutional context here refers to the formal and informal rules, norms, logics and mandates that shape actor roles, incentives and expectations. Across interviews and observations, we found that KT took on different meanings, practices and forms on the basis of what was institutionally possible, legitimate or incentivized. For example, researchers operate within institutions where the dominant logic is that of producing evidence through time-bound, funder driven projects. As described earlier in the findings, KT in these settings was often structured, technical and bounded to research projects (structured KT). This reflected the institutions’ framing of what researchers are responsible for. By contrast, multilateral institutions spanned multiple logics, for example, technical, programmatic and so on, which enabled them to participate closely throughout the policy process. This institutional position and access to funding allowed for fluid KT and conferred these actors with both formal authority and informal influence.

#### Politico-economic context

The politico-economic context determined the types of evidence that were seen as legitimate, actionable or even relevant in policy-making. Three illustrative examples demonstrate this. First, Kenya’s decentralized governance structure shaped divergent evidence needs. At the national level, where decisions were made for the entire country, policy-makers often relied more on large-scale data (for example, national surveys) to support policy formulation. By contrast, county level policy-makers whose main responsibility was service delivery, relied on operational data to inform policy decisions. This reflects the distinct roles each level plays in the devolved health system:

“So, decision-making at that [service delivery] level is informed by evidence of staff deployment, rationalisations, it is also informed by evidence of how big the health facility is, how many service delivery sites it has, size of the workload, the catchment area one is serving, the morbidity pattern of that region…” County policy-maker 002

Second, for politically charged issues, the selection and use of evidence was often strategic. Evidence that aligned with political interests was favoured while evidence that conflicted with these interests was ignored:

“They [legislators] don’t take the options you have given them, and they have a different option that is not based on evidence but based on their party inclination which is not … their option doesn’t have a rationale except the fact that it is what the party requires.” Researcher 006

Third, in a context of limited fiscal space, even robust evidence was unlikely to be considered if its application was perceived as financially or operationally unfeasible. Policy-makers often described economic constraints as a filter which narrowed the evidentiary landscape to only what could be realistically acted upon:

“You know you can have a lot of evidence but there are just simply no finances to translate that to action.” National policy-maker 004

Political and economic dynamics thus operated as causal mechanisms that activated or constrained KT by determining when evidence could be mobilized, by whom and for what purposes.

#### Structural–operational context

The structural–operational context actively produced the spaces, opportunities and constraints through which KT was practised. First, the devolved governance structure expanded the landscape for KT creating a new layer of actors, decisions and introduced new the spaces where actors could choose to engage:

“In fact, we’ve pivoted a lot of our work and support to working with counties [from national government] because when you have the right county, the right leadership, you can change so much in a very short time” Development partner 008

Second, efforts to institutionalize evidence use in policy-making led to establishment of KT and research units at the MoH and the development of national evidence-informed policy-making guidelines. These institutional arrangements formalized KT roles and processes offering spaces for coordination and routine engagement:

“Yes, since we developed the [research] unit, now we have identified our areas of research, needs of research we have identified we have done analysis and identified them.” County Policy-maker 005

All together, these findings demonstrate how context coproduces KT by structuring who has influence, what counts as evidence and where and how KT happens. In this sense, KT both reflects and coproduces the political and institutional realities of the health policy system.

### What are the outcomes of KT?

The KT outcomes observed in this study were incremental and relational in nature. However, there were instances where evidence directly influenced a decision such as budgeting, identifying priority interventions or adjusting existing plans. These cases often involved relatively technical/planning decisions where the evidence was seen as actionable and timely:

“Across all these four counties, in less than 2 years, budget execution has improved to between 90% and 100% in all four counties [after KT intervention].” Development partner 008

KT outcomes also included shaping opinions and building consensus among stakeholders. For example, actors used benchmarking studies or implementation experiences from other settings to persuade sceptics or soften opposition. These KT efforts aimed to align diverse interests around a shared narrative or policy goal:

“We took very many counties to [county x] who are trying to implement these facility autonomy reforms for them to actually see how things could work or how things are working in other counties.” Development partner 008

In several cases, participants described institutional shifts, including the integration of evidence-informed practices into standard operating procedures. A good example of this is the institutionalization of health technology assessments when designing the national health benefits package.

Participants also described strengthened relationships between actors, particularly where engagement was sustained. In many cases, these relationships opened doors for future evidence contributions, which participants framed as a key enabler for policy influence:

“I think successes in that we’ve managed to maintain, I like to think reasonably good working relationships for 25 years through very many rounds of changes in the Ministry of Health” Researcher 001

## Discussion

Our findings show how KT is constituted through context and shaped by the interaction of actors, the forms of KT they practice and the types of evidence they use. Ultimately, what KT for policy looks like is a product of the political, institutional and structural conditions in which it happens.

Our study informs the reframing of KT-policy in two main ways. First, it reconceptualizes context as a constitutive force that produces the design, form and possibilities of KT. We show that actors’ motivations for doing KT, how they did it and where, are all embedded in specific institutional, political and social contexts that define what is possible and actionable. These findings support arguments that emphasize context as dynamic and coproductive of KT and goes further by illustrating empirically how context produces what is considered legitimate knowledge, who gets to participate in KT and to what ends [30, 40, 41].

Second, it reframes KT practice from a technocratic one to a diffuse and situated practice. While in much of KT literature, KT is conceptualized as dynamic and context-specific, it is still approached as a technical process, defined by discrete steps which are often formalized through strategies, interventions or toolkits [42–45]. This scholarship accommodates complexity but still treats KT as something that can be planned, managed and delivered. In contrast, this study finds that KT is also practised as a fluid, embedded and situated practice that is shaped by actors’ positionalities, institutional logics and political dynamics. This aligns with literature that emphasizes the relational, situated, iterative and political nature of KT [17, 46, 47]. In particular, Evans and Scarbrough (2014) described KT processes in healthcare organizations as ranging from discrete, episodic ‘bridging’ to embedded, relational ‘blurring’ – a distinction that resonates with this study’s finding of structured and fluid KT forms [48]. Furthermore, the idea of KT processes existing on a spectrum between more structured, instrumental practices and more embedded, relational forms resonates with the K* spectrum/concept which captures the diversity of KT related practices across research–policy interfaces [49]. Similarly, we show that KT is embedded in social relationships and networks. For example, the fluid forms of KT practice which several actors enact and the informal spaces where they sometimes happened relied on informal relationships, trust-based exchanges and repeated interaction. These social structures enabled more adaptive forms of KT in our setting. This is consistent with the work by Contandriopoulos et al. (2010) and others, which have shown that trust, credibility and positionality shape whose evidence is heard and acted upon [17, 19, 31, 50–52].

These shifts in KT framing pose significant implications for both KT theory and practice. For theory, they call for a shift toward practice-based and relational models of KT. Such a move means that attention needs to be paid to the everyday negotiations, institutional routines and policy work through which evidence is mobilized. Similarly, viewing context as constitutive of KT means that KT theory would need to address how actors’ power, position and institutional structures produce KT and its outcomes. For practice, this reframing highlights the importance of building relationships, navigating institutional logics and working reflexively within the evidence-policy system. For KT practitioners, this requires an ongoing, adaptive engagement with the policy environment and with the actors who shape it. Importantly, these insights also signal that capacity-building efforts must go beyond training in technical skills to support relational, strategic and systems-level competencies.

## Strengths and limitations

The conceptual framework offers a way of examining how KT is constituted within policy processes across different institutional settings. While the specific dynamics observed here are grounded in the Kenyan health system, the framework provides a conceptual lens that may be useful for analysing KT in other policy environments. In addition, by drawing on rich, context-sensitive qualitative data across multiple actors, data sources and institutional settings, the study was able to generate nuanced insights into the realities of KT in health policy contexts. By focusing on health policies in general, the study captured cross-cutting patterns in KT. This allowed us to highlight the systemic conditions that produce or inhibit KT rather than the idiosyncrasies of a single policy process. This approach lent itself to our aim of conceptual reframing because it revealed patterns that transcend specific policy issues.

However, the absence of focused tracer policies limited our ability to examine how KT happens over time in relation to specific decisions. In addition, given the closed and hierarchical nature of policy-making, access to several key spaces and actors was restricted.

## Conclusions

Together, these findings reframe KT as a diffuse, situated and political practice that is produced by context. Engaging more critically with this reframing offers the potential to practice KT that is more meaningful, responsive and sustainable in policy settings.

## Supplementary Information

Supplementary Material 1.

## Data Availability

The data underlying this article are not publicly available as that would breach ethics protocol for this study. De-identified excerpts of the transcripts relevant to the study can be available upon reasonable request to the corresponding author.

## Abbreviations

KT: Knowledge translation
STS: Science and technology studies
iKT: Integrated knowledge translation
EIPM: Evidence-informed policy-making
TWG: Technical working group
NGO: Nongovernmental organization
MoH: Ministry of Health
CDoH: County department of health
HMIS: Health management information system
LMIC: Low–middle income countries
RCT: Randomized control trial
CHMT: County health management team
SAGA: Semi-autonomous government agency
KEMRI: Kenya Medical Research Institute
KWTRP: KEMRI-Wellcome Trust Research Programme
SERU: Scientific ethics review unit
FIF: Facility improvement fund
HRH: Human resource for health
HR: Human resource
WHO: World Health Organization
PHC: Primary health care
EtD: Evidence to decision framework
UHC: Universal health coverage
SHA: Social health authority
PPB: Poisons and pharmacy board
KHPOA: Kenya Health Professions Oversight
KMA: Kenya Medical Association
KUCO: Kenya Union of Clinical Officers
LVCT: Liverpool Voluntary Counselling Center
KHDS: Kenya Health Demographic Survey
HTA: Health technology assessment

## Declarations

### Reflexivity statement

The research team comprised two women and two men, all working in low- and middle-income country settings. The lead author is a PhD student with prior experience in policy-engaged research, and the three co-authors were senior researchers with established expertise in health systems and qualitative methods. The team’s positionalities, across gender, career stage and professional background, shaped how the study questions were framed and how data were interpreted. The authors’ familiarity with LMIC policy environments facilitated access to participants and informed the analytic lens, while ongoing discussion within the team was used to reflect on assumptions, mitigate individual biases and ensure that multiple interpretations were considered throughout the study.
