# Supplementary material for: Reframing knowledge translation for health policy in Kenya: actors, practices and the constitutive role of context
Source: Health Res Policy Syst. 2026 Apr 19;24:47. doi: 10.1186/s12961-026-01482-5 (PMC13224559; doi:10.1186/s12961-026-01482-5)
Supplement: Supplementary file 1 — Supplementary Material 1. [file 12961_2026_1482_MOESM1_ESM.docx]

Supplementary table 1: In-depth interview guides

| **Construct** | **Key interview points** |
| --- | --- |
| Context | - Political, social, economic, organisational, or interpersonal factors that were present at the time of engagement/ knowledge production/policy making that may have affected KT outcomes. |
| Policy issue | - How are policy issues defined and perceived by actors (how does an issue become a policy issue? who decides what a policy issue is?) - Characteristics of issues that facilitate/hinder knowledge use |
| Actors | - Actor identities & roles (who they are and what their roles are) - Actor characteristics/qualities that impact KT and research use - Their impact on KT outcomes (champions, blockers etc) |
| Knowledge | - Type and sources of knowledge considered for policy (explore perceptions of these knowledge types) - How actors perceive knowledge e.g particular findings and how that may affect use (explore how contested knowledge is dealt with between actors) - Characteristics of knowledge that influence use |
| Knowledge translation processes/activities/interventions | - Description of KT processes/activities etc. (what knowledge is being translated, by whom, to whom, how, when) - Experiences and perceptions of KT processes with regards to how it facilitates learning and discussion between actors |
| KT Outcomes | - Impact of KT processes/activities (how impact is defined and measured) - How have KT processes affected:  1. Actor relationships and characteristics with relation to uptake of research 2. Learning and mutual understanding between actors 3. Policy outcomes (instrumental use)  - What contributed to success/failure to achieve impact |

Supplementary table 2: Observations and document review guides

| Data collection method | Guide |
| --- | --- |
| Non-participant observations checklist | 1. What is the goal of the meeting/engagement? 2. Who is present? What formal roles/affiliations do they hold? 3. Who initiated the engagement? 4. Who is funding it? 5. Where is the engagement taking place? 6. What is the tone of interaction (collaborative, neutral etc) 7. Is there alignment/conflict between actors 8. Who is dominating/leading the discussions? 9. What evidence/arguments are being presented/emphasised? 10. Are decisions being made? Which? How? 11. Is evidence used to inform the decision? Which type? How? |
| Document review checklist | 1. What is the type of document? 2. Who authored/what institution is it affiliated with 3. What is the stated purpose of the document? 4. Who are the key actors mentioned in the document? 5. What are their roles? 6. What types of evidence are included? |

Supplementary table 3: List of documents reviewed

| **Types of documents** | **Examples** |
| --- | --- |
| Campaign manifesto | - The Kenya Kwanza Plan: The bottom-up economic transformation agenda (2022-2027) |
| Government documents (laws, policies, strategies and guidelines | - Public Policy Handbook for Kenya - Intergovernmental Relations Act (no 2 of 2012) - Kenya Health Sector Strategic Plan (2023-2027) - Kenya Health Sector Partnership and Coordination plan - Sector working group report: Medium Term Expenditure Framework (MTEF) for the period 2023 - Kenya Health Policy 2014-2030 - Kenya Universal Health Coverage Policy 2020-2030 - Kenya Community Health policy 2020-2030 - Kenya Primary Health Care Strategic Framework (2019 – 2024) - Kenya Health Sector Monitoring & Evaluation Plan 2019 - Ministry of Health Organogram - Kenya Health financing strategy 2020-2030 - Guidelines for Evidence Use in Policy-making 2016 |
| Local academic and research organisations | - Report of the Baseline Survey on the Context and Status of Research Use in Policy Formulation at the Ministry of Health in Kenya (2016) - Reports on stakeholder engagement workshops - PowerPoint presentations made during stakeholder workshops |
